# Supplementary material for: Decompression and Interlaminar Stabilization for Lumbar Spinal Stenosis: A Cohort Study and Two-Dimensional Operative Video
Source: Medicina (Kaunas). 2022 Apr 5;58(4):516. doi: 10.3390/medicina58040516 (PMC9031522; doi:10.3390/medicina58040516)
Supplement: Supplementary file 1 [file medicina-58-00516-s001.zip › Table S1.pdf]

Table S1: Cohort Datasheet

| | | VAS Scores | | | | | | | | ODI Scores | | | |
|---|---|---|---|---|---|---|---|---|---|---|---|---|---|
| | | Low Back | | | | Lower Extremities | | | | | | | |
| **Age** | **Levels** | **Pre- op** | **2 mo** | **6 mo** | **12 mo** | **Pre-op** | **2 mo** | **6mo** | **12 mo** | **Pre-op** | **2 mo** | **6 mo** | **12 mo** |
| 80 | 45 | 10 | 5 | 5 | 4 | 10 | 3 | 3 | 3 | 73 | 32 | 30 | 28 |
| 70 | 45 | 9 | 5 | 4 | - | 10 | 4 | 4 | - | 66 | 23 | 21 | - |
| 59 | 45 | 8 | 4 | 3 | - | 8 | 2 | 2 | - | 62 | 24 | 20 | - |
| 80 | 234 | 10 | 5 | 5 | 4 | 10 | 5 | 4 | 4 | 74 | 35 | 33 | 30 |
| 74 | 345 | 9 | 4 | 3 | 3 | 9 | 2 | 2 | 2 | 57 | 23 | 24 | - |
| 87 | 34 | 10 | 5 | 4 | 4 | 10 | 2 | 2 | 2 | 77 | 32 | 28 | 27 |
| 57 | 45 | 9 | 4 | 4 | - | 9 | 2 | 2 | - | 69 | 25 | 24 | - |
| 71 | 24 | 9 | 5 | 4 | 3 | 8 | 3 | 3 | 3 | 66 | 21 | 20 | 20 |
| 76 | 35 | 8 | 5 | 4 | 5 | 10 | 4 | 3 | 3 | 75 | 28 | 30 | 32 |
| 61 | 45 | 7 | 3 | 3 | 2 | 7 | 2 | 2 | 2 | 58 | 16 | 12 | 13 |
| 64 | 35 | 8 | 3 | 3 | 2 | 9 | 3 | 2 | 2 | 63 | 18 | 17 | 15 |
| 70 | 45 | 8 | 2 | 5 | 8 | 8 | 2 | 4 | 5 | 57 | 15 | 25 | 48 |
| 75 | 345 | 9 | 3 | 2 | 2 | 9 | 2 | 2 | 2 | 70 | 18 | 20 | 19 |
| 60 | 45 | 8 | 3 | 2 | 3 | 8 | 2 | 2 | 2 | 57 | 12 | 10 | 13 |
| 53 | 34 | 8 | 2 | 3 | 4 | 7 | 2 | 2 | 2 | 58 | 16 | 18 | 22 |
| 63 | 345 | 9 | 3 | 4 | 6 | 9 | 2 | 2 | 2 | 68 | 18 | 25 | 28 |
| 57 | 34 | 10 | 6 | 6 | 6 | 9 | 4 | 4 | 3 | 72 | 38 | 37 | 34 |
| 62 | 234 | 10 | 6 | 5 | - | 10 | 3 | 3 | - | 74 | 35 | 30 | - |
| 49 | 45 | 9 | 5 | 5 | - | 9 | 3 | 2 | - | 71 | 30 | 28 | - |
| 62 | 45 | 8 | 2 | 1 | 1 | 9 | 2 | 1 | 1 | 64 | 15 | 10 | 10 |
